# Application of a complete blood count to screening lethargic and anorectic cats for pancreatitis

**DOI:** 10.1186/s12917-021-03098-z

**Published:** 2021-12-11

**Authors:** Magdalena Maria Krasztel, Michał Czopowicz, Olga Szaluś-Jordanow, Agata Moroz, Marcin Mickiewicz, Jarosław Kaba

**Affiliations:** 1 Division of Veterinary Epidemiology and Economics, Institute of Veterinary Medicine, Warsaw University of Life Sciences-SGGW, Nowoursynowska 159c, 02-776 Warsaw, Poland; 2 Department of Small Animal Diseases with Clinic, Institute of Veterinary Medicine, Warsaw University of Life Sciences-SGGW, Nowoursynowska 159c, 02-776 Warsaw, Poland

**Keywords:** Abdominal ultrasonography, Band neutrophil count, Eosinophil count, Feline pancreatic lipase immunoreactivity, fPLI, Neutrophil-to-lymphocyte ratio, White blood cell count

## Abstract

**Background:**

Feline pancreatitis (FP) is an important health problem of cats. Its diagnostics is based on the combination of quantification of serum pancreatic lipase immunoreactivity (fPLI) and abdominal ultrasonography (AUS). These modalities allow for establishing highly specific diagnosis, however they are relatively expensive and time-consuming. On the other hand, a screening test of high sensitivity which would allow to rule out FP on the first visit without a considerable increase of costs would be clinically useful. To evaluate accuracy of nonspecific inflammatory biomarkers based on complete blood count (CBC) in diagnosing FP 73 client-owned cats with signs of lethargy and reduced appetite lasting for at least 2 days before presentation were enrolled in the cross-sectional study. They were examined with fPLI assay and AUS and classified as cats with very low risk of FP when fPLI ≤3.5 μg/L and AUS negative for FP, or as cats with increased risk of FP in the case of any other combination of results. Then, 7 various CBC measurements were measured in each cat and linked to the risk of FP using the multivariable logistic regression.

**Results:**

Five CBC measurements turned out to be significantly associated with the risk of FP – total leukocyte count (WBC; crude odds ratio(OR_crude_) = 12.2; CI 95%: 1.52, 98.5), total neutrophil count (OR_crude_ = 5.84; CI 95%: 1.22, 27.9), band neutrophil count (BNC; OR_crude_ = 6.67; CI 95%: 1.98, 22.4), neutrophil-to-lymphocyte ratio (OR_crude_ = 3.68; CI 95%: 1.25, 10.9), and eosinophil count (EC; OR_crude_ = 0.34; CI 95%: 0.12, 0.96). The model based on WBC, BNC, and EC proved to have at least fair diagnostic potential (area under ROC curve 82.7%; CI 95%: 72.8%, 92.5%). When WBC <  18 G/L, BNC <  0.27 G/L, and EC >  0.3 G/L was considered as a negative result, and any other combination as the positive result, the CBC model had high sensitivity (91.8%; CI 95%: 80.8%, 96.8%) at a relatively low specificity (58.3%; CI 95%: 38.8%, 75.5%).

**Conclusion:**

The combination of three CBC measurements is an immediately available and fairly accurate screening method for identification of lethargic and anorectic cats with increased risk of FP.

**Supplementary Information:**

The online version contains supplementary material available at 10.1186/s12917-021-03098-z.

## Background

Feline pancreatitis (FP) is a relatively common disease whose etiology usually remains unknown, clinical manifestation is nonspecific, diagnostics difficult and expensive, and prognosis guarded [1–5]. Lethargy and reduced appetite or anorexia are most common clinical signs, followed by vomiting, weight loss, and less often diarrhea [5]. Even though, histologically three different FP forms are recognized – acute necrotizing pancreatitis, acute suppurative pancreatitis, and chronic pancreatitis [6, 7], their similar clinical manifestation renders them barely distinguishable for a practitioner [8]. Therefore, the general term feline pancreatitis is commonly used [5].

At present the combination of clinical chemistry assays measuring pancreatic lipase immunoreactivity (serum pancreatic lipase immunoreactivity, fPLI) or activity (colorimetric 1,2-o-Dilauryl-Rac-Glycero-3-Glutaric Acid-(6′-Methylresorufin) Ester (DGGR) assay) and abdominal ultrasonography (AUS) is a mainstay of clinical diagnostics of FP [2, 3, 5, 9]. The estimates of diagnostic accuracy of these tests vary between studies. Diagnostic sensitivity (Se) and specificity (Sp) range from 50% to 80% and from 50% to 100%, respectively, for fPLI [10–14] and from 25% to 80% and from 70% to 90%, respectively, for AUS [10, 15, 16]. These discrepancies result from different cut-off values of the test (fPLI of 3.5 or 5.3 μg/L; 1 or 2 ultrasonographic changes indicative of FP), different severity of FP in a study population (mild or marked) as well as differences in the skills of AUS examiners and quality of ultrasound devices. Nevertheless, most of studies indicate that Sp outweighs Se [2, 3, 17]. Hence, unless a pre-test probability of FP is very low, positive results of these tests tend to be more trustworthy than negative (i.e. positive predictive value, PPV outraces negative predictive value, NPV). This renders them rather confirmatory then screening tests. Therefore, an easily available and inexpensive screening method of high Se for an immediate identification of patients with increased risk of FP would be of value.

Various inflammatory biomarkers are used in human and veterinary medicine for tentative detection, or rather exclusion, of active inflammatory, infectious and neoplastic processes [18]. Of them biomarkers based on complete blood count (CBC) such as total (WBC) and differential leukocyte count, as well as neutrophil-to-lymphocyte ratio (NLR) have been investigated in small animal veterinary medicine mainly due to their low cost and broad availability [19–23]. The presence or severity of pancreatitis in cats have been recently linked to NLR [22].

In this study, we evaluated the accuracy of a handful of CBC measurements in making the first-line decision whether a cat with lethargy and reduced appetite was at increased risk of FP.

## Results

The study enrolled 73 adult castrated cats, 45 males (61.6%) and 28 females (38.4%), aged from 2 to 18 years with the median (IQR) of 10 (8 to 12) years. Age did not differ significantly between sexes (*p* = 0.750). Fifty eight cats (79.5%) were domestic shorthair. Others were Siberian (*n* = 5, 6.9%), British shorthair (*n* = 3, 4.1%), Maine coon, Russian and Devon rex (each *n* = 2, 2.7%), and Siamese (*n* = 1, 1.4%).

fPLI was normal (≤ 3.5 μg/L) in 33 cats (45.2%) and elevated (> 3.5 μg/L) in 40 cats (54.8%): from 3.6 to 5.3 μg/L in 8 cats and > 5.3 μg/L in 32 cats. At least one ultrasonographic feature of FP was detected in 26 cats (35.6%). On the basis of those two diagnostic tests the risk of FP was determined as very low (fPLI ≤3.5 μg/L and normal appearance of the pancreas in AUS) in 24 cats (32.9%) and increased (fPLI > 3.5 μg/L and/or abnormal appearance of the pancreas in AUS) in 49 cats (67.1%). From among 49 cats with increased risk of FP only 17 cats (34.7%) had both elevated fPLI and abnormal appearance of the pancreas in AUS. Twenty three cats (46.9%) had elevated fPLI but normal appearance of the pancreas in AUS, and 9 cats (18.4%) had abnormal appearance of the pancreas in AUS but normal fPLI.

Diabetes mellitus was present in 29 cats (39.7%), suspected hepatopathy in 22 cats (30.1%; ALT [median (range)] – 307 (58–2322) U/L; TB – 18.8 (6.8–359.1) μmol/L), suspected acute kidney injury in 7 cats (9.6%; urea – 29.0 (17.9–62.7) mmol/L, creatinine – 353.6 (256.4–1246) μmol/L), and neoplastic disease in 3 cats (4.1%) (malignant liver tumor, intestinal lymphoma, and disseminated pulmonary metastases from the mammary gland tumor). Hemoconcentration was observed in 23 cats (31.5%; Ht – 0.38 (0.16–0.51), TP – 73 (52–97) g/L, urea – 19.9 (9.1–97.2) mmol/L, creatinine – 168 (97.2–256.4) μmol/L) and anemia in 10 cats (13.7%; Ht – 0.24 (0.16–0.26)). Vomiting was reported on admission in 37 cats (50.7%) and diarrhea in 9 cats (12.3%). Abdominal pain was detected by an attending veterinarian in 13 cats (17.8%) and fever in 3 cats (4.1%). Cats with increased risk of FP were significantly older (*p* = 0.038). All other demographic and clinical characteristics were evenly distributed between cats with very low and increased risk of FP (Table 1).

**Table 1:** Demographic and clinical characteristics of the study cats

Characteristic^a^	Risk of feline pancreatitis		*P*-value^b^
	Very low (*n* = 24)	Increased (*n* = 49)	
Demographic characteristics			
Age [years] – median, IQR, (range)	10, 7–11 (2–15)	11, 9–13 (2–18)	0.038*
Sex – males	14 (58.3)	31 (63.3)	0.685^c^
Breed – domestic shorthair	3 (12.5)	12 (24.5)	0.218
Comorbidities			
Diabetes mellitus	9 (37.5)	20 (40.8)	0.785
Suspected hepatopathy	5 (20.8)	17 (34.7)	0.216
Suspected acute kidney injury	2 (8.3)	5 (10.2)	0.999^d^
Anemia	2 (8.3)	8 (16.3)	0.481^d^
Hemoconcentration	6 (25.0)	17 (34.7)	0.397
Hyperthyroidism	1 (4.2)	0	0.319^d^
Neoplastic disease	0	3 (6.1)	0.546^d^
Clinical signs			
Vomiting	9 (37.5)	28 (57.1)	0.113
Diarrhea	6 (25.0)	3 (6.1)	0.051^d^
Abdominal pain	3 (12.5)	10 (20.4)	0.407
Fever	0	3 (6.1)	0.546^d^

^*^Significant at α = 0.05

^a^Presented as n(%) unless otherwise stated

^b^Maximum likelihood G-test unless otherwise stated

^c^Mann-Whitney U test

^d^Fisher exact test

Numerical values of 7 CBC measurements and cut-off values used for their categorization are presented in Additional file 1.

The univariable analysis yielded 5 CBC measurements significantly linked to an increased risk of FP – four positively associated: WBC, total neutrophil count (TNC), band neutrophil count (BNC), NLR, and one negatively associated: eosinophil count (EC) (Table 2).

**Table 2:** The univariable analysis of the association between categorized complete blood count (CBC) measurements and increased risk of feline pancreatitis (FP) in the study cats

CBC-based inflammatory biomarker	Category	Cats with increased risk of FP / all cats in the category (%)	*P*-value^a^	Crude odds ratio (OR_crude_) (CI 95%)
Total leukocyte count [G/L]	≥ 18 (*n* = 18)	17 (94.4)	0.002*	12.2 (1.52, 98.5)
	< 18 (*n* = 55)	32 (58.2)		
Total neutrophil count [G/L]	≥ 15 (*n* = 19)	17 (89.5)	0.010*	5.84 (1.22, 27.9)
	< 15 (*n* = 54)	32 (59.3)		
Band neutrophil count [G/L]	≥ 0.27 (*n* = 32)	28 (87.5)	0.001*	6.67 (1.98, 22.4)
	< 0.27 (*n* = 41)	21 (51.2)		
Lymphocyte count [G/L]	≥ 2.2 (*n* = 36)	21 (58.3)	0.113	0.45 (0.17, 1.23)
	< 2.2 (*n* = 37)	28 (75.6)		
Eosinophil count [G/L]	> 0.3 (*n* = 36)	20 (55.6)	0.037*	0.34 (0.12, 0.96)
	≤ 0.3 (*n* = 37)	29 (78.4)		
Monocyte count [G/L]	≥ 0.15 (*n* = 12)	11 (91.6)	0.089	6.95 (0.84, 57.3)
	< 0.15 (*n* = 61)	38 (62.3)		
Neutrophil-to-lymphocyte ratio [1/1]	≥ 4.7 (*n* = 33)	27 (81.8)	0.013*	3.68 (1.25, 10.9)
	< 4.7 (*n* = 40)	22 (55.0)		

*Significant at α = 0.05

^a^The maximum likelihood G-test

Three CBC measurements proved to be significantly associated with increased risk of FP in the multivariable analysis (Table 3). The odds of being at increased risk of FP were 13-fold higher when WBC was ≥18 G/L and almost 5-fold higherwhen BNC was ≥0.27 G/L, while they were roughly 4-fold lower when EC was > 0.3 G/L. The model based on 3 CBC measurements (henceforth referred to as CBC model) fit the data well (H&L χ^2^ = 3.04, *p* = 0.551; Nagelkerke’s pseudo-*R*^2^ = 0.40).

**Table 3:** Multiple logistic regression model based on a complete blood count (CBC) aiming to identify these anorectic and lethargic cats in which the risk of pancreatitis is increased

CBC measurements	Regression coefficient (SE)	Wald’s statistics	*P*-value	OR_adj_ (CI 95%)
Intercept	0.52 (0.46)	–	–	–
WBC ≥ 18 G/L	2.59 (1.12)	5.32	0.021	13.3 (1.47, 121)
BNC ≥ 0.27 G/L	1.59 (0.67)	5.56	0.018	4.88 (1.31, 18.2)
EC > 0.3 G/L	−1.49 (0.61)	5.98	0.014	0.23 (0.07, 0.74)

*BNC* Band neutrophil count, *CI 95%* 95% confidence interval, *EC* Eosinophil count [G/L], *OR*_*adj*_ Adjusted odds ratio, *SE* Standard error, *WBC* Total leukocyte count [G/L]

**Fig. 1:**
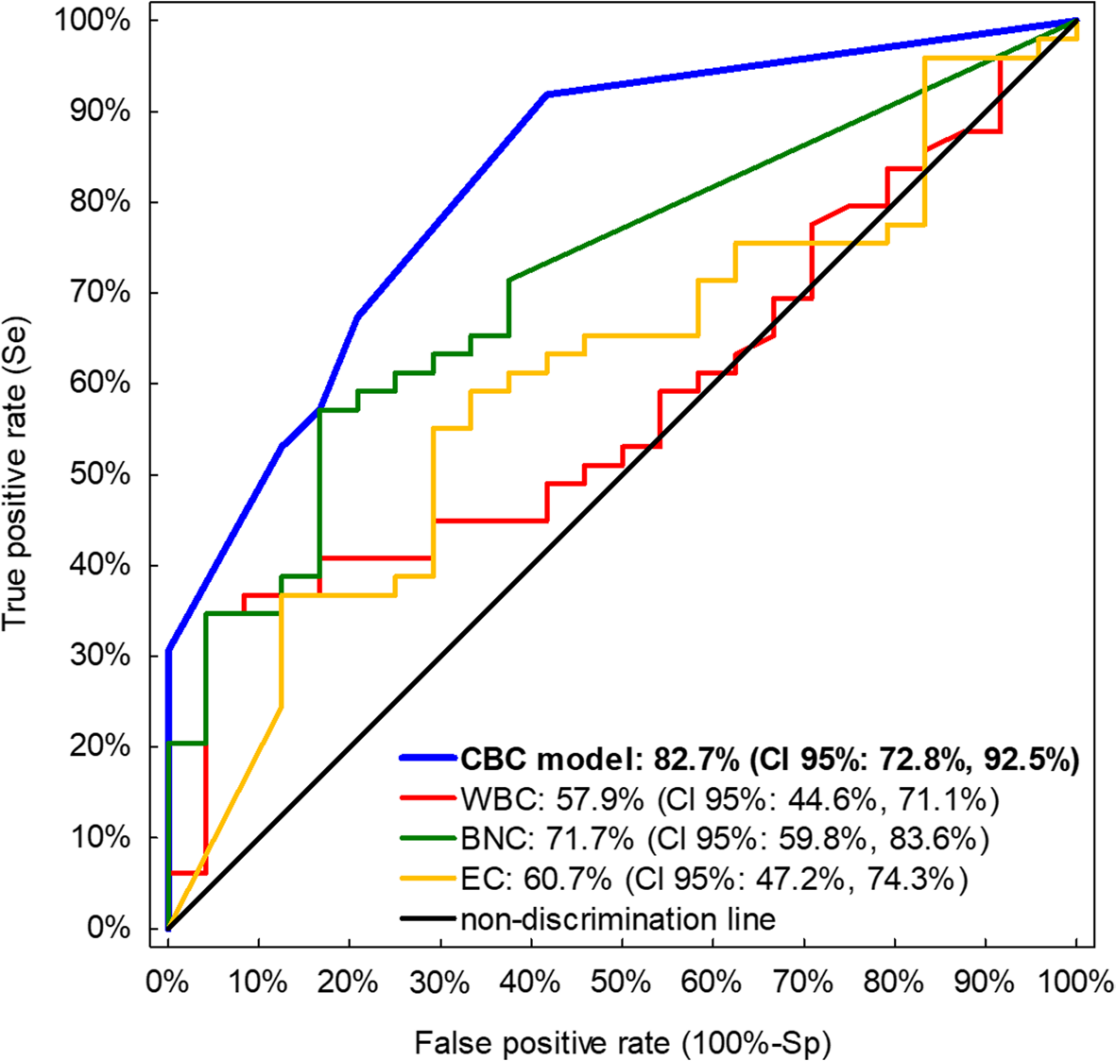
ROC curves of the logistic model developed using three complete blood count (CBC) measurements as well as of each CBC measurement: WBC – total leukocyte count [G/L], BNC – band neutrophil count [G/L], EC – eosinophil count [G/L]). Area under ROC curve with 95% confidence interval (CI 95%) presented for each CBC measurement

The CBC model had fair to good discriminatory potential (AUROC = 82.7%; CI 95%: 72.8%, 92.5%; *p* <  0.001), which was significantly higher than a discriminatory potential of each CBC measurement separately (Fig. 1): mean difference of AUROC between the CBC model and: WBC = 24.8% (CI 95%: 10.9%, 38.7%; *p* = 0.001); BNC = 11.0% (CI 95%: 1.6%, 20.4%; *p* = 0.022); EC = 22.0% (CI 95%: 7.4%, 36.3%; *p* = 0.003).

At the optimal cut-off value ≥0.628 (i.e. result of the CBC model considered as negative only when WBC <  18 G/L, BNC <  0.27 and EC >  0.3 G/L) the CBC model’s Se was 91.8% (CI 95%: 80.8%, 96.8%), and Sp was 58.3% (CI 95%: 38.8%, 75.5%), while positive (LR+) and negative (LR-) likelihood ratios were 2.2 (CI 95%: 1.4, 3.6) and 0.14 (CI 95%: 0.05, 0.38), respectively. The accuracy measures of the CBC model at other cut-off values (i.e. at other combinations of the three CBC measurements included in the CBC model) are presented in Table 4.

**Table 4:** Accuracy of complete blood count (CBC) model at various cut-off values i.e. at various combinations of CBC measurements included in the model. The optimal cut-off value determined on the basis of the highest Youden’s index (J) highlighted (the combination above it i.e. WBC < 18 G/L, BNC < 0.27 G/L, and EC > 0.3 G/L is the only to be considered as negative)

Combinations of CBC measurements			Result of CBC model	Accuracy measures				
WBC ≥ 18	BNC ≥ 0.27	EC > 0.3		Se (CI 95%)	Sp (CI 95%)	LR+ (CI 95%)	LR- (CI 95%)	J
No	No	Yes	0.275	–	–	–	–	–
**No**	**No**	**No**	**0.628**	**91.8 (80.8, 96.8)**	**58.3 (38.8, 75.5)**	**2.2 (1.4, 3.6)**	**0.14 (0.05, 0.38)**	**50.2 (39.4, 61.0)**
No	Yes	Yes	0.649	67.3 (53.4, 78.8)	79.2 (59.5, 90.8)	3.2 (1.4, 7.2)	0.41 (0.26, 0.65)	46.5 (35.9, 57.2)
Yes	No	Yes	0.835	57.1 (43.3, 70.0)	83.3 (64.1, 93.3)	3.4 (1.4, 8.7)	0.51 (0.36, 0.74)	40.5 (30.1, 50.9)
No	Yes	No	0.892	53.1 (39.4, 66.3)	87.5 (69.0, 95.7)	4.2 (1.4, 12.6)	0.54 (0.38, 0.75)	40.6 (30.7, 50.4)
Yes	No	No	0.957	30.6 (19.5, 44.5)	100 (86.2, 100)	+∞	0.69 (0.58, 0.84)	30.6 (24.0, 37.2)
Yes	Yes	Yes	0.961	24.5 (14.6, 38.1)	100 (86.2, 100)	+∞	0.76 (0.64, 0.89)	24.5 (18.3, 30.6)
Yes	Yes	No	0.991	6.1 (2.1, 16.5)	100 (86.2, 100)	+∞	0.94 (0.87, 1.01)	6.1 (2.7, 9.5)

*BNC* Band neutrophil count [G/L], *CI 95%* 95% confidence interval, *EC* Eosinophil count [G/L], *J* Youden’s index [%], *LR+* Likelihood ratio of a positive result [1/1], *LR-* Likelihood ratio of a negative result [1/1], *Se* Diagnostic sensitivity [%], *Sp* Diagnostic specificity [%], *WBC* Total leukocyte count [G/L]

## Discussion

In this study we developed a 3-element logistic regression model (CBC model) which has at least fair accuracy (confirmed by the lower 95% confidence limit above the threshold of 70%) in distinguishing between symptomatic cats with very low or increased risk of FP. The CBC model has two important upsides. First, it is based on basic hematologic measurements (CBC-based inflammatory biomarkers: WBC, BNC, and EC) which are routinely examined in virtually all apparently sick patients and are available from most veterinary laboratories within a few hours of blood collection. Secondly, CBC measurements were included in the CBC model as dichotomous variables categorized according to a cut-off value which was determined using sound statistical methodology (Youden’s index-based criterion) [24, 25]. Three binary variables result in only 2^3^ = 8 potential combinations. As a result no calculations are necessary to apply the CBC model in daily practice. The only essential thing is the knowledge whether a given CBC measurement is below or above the cut-off value (Table 4).

CBC model is based on three types of leukocytes whose categorization corresponds to three well recognized changes observed in leukogram during the acute phase response: leukocytosis, left-shift, and eosinopenia [26–28]. Leukocytosis usually results from neutrophilia which develops easily and quickly in cats due to relatively high ratio (3:1) of marginal to circulating pool of neutrophils [26, 27, 29]. Immature band neutrophils are released from the bone marrow storage pool only when the inflammatory process leading to excessive migration of neutrophils to the affected tissues lasts for a longer time [26, 29]. The fact that our study enrolled cats in which nonspecific clinical signs had been observed for at least 2 days explains important role of the left-shift in identifying cats with FP. However, it is possible that this feature may not be equally important if cats in which symptoms have begun more recently are being examined. In humans and many animal species neutrophilia is typically accompanied by lymphopenia during the acute inflammatory reaction [30]. In cats, however, it is not rare to observe the elevation of both neutrophils and lymphocytes [27, 28], especially when a concurrent excitement response triggered by stressful events such as physical examination, restraint and blood collection occur. In cats marked lymphocytosis is a prominent feature of the excitement response [26, 29, 31]. This may to some extent explain why lymphopenia was not included in the model and why NLR, which captures both neutrophilia and lymphopenia at the same time and therefore shows diagnostic usefulness in humans [32] and, to some extent, in dogs [19, 20], performed inferior to WBC in our study.

Our study aimed to identify CBC measurements which can be useful in making a tentative diagnosis of FP. Obviously, these measurements are not linked specifically to the pathological processes developing in the course of FP so they cannot be expected to perform as a highly accurate confirmatory test. In fact they may play role only in an immediate exclusion of FP, so they may work as a rapid screening test. However, to satisfy this expectation they must have sufficiently high Se so that a practitioner may thrust their negative result. Se of the CBC model at an optimal cut-off value was roughly 90%, or at least 80% when we refer to the lower 95% confidence limit. Even though, it may appear to be quite a high figure, we should focus on NPV, or more conveniently, on LR- which is independent of disease prevalence in a population [25]. LR- for CBC model was roughly 0.14. It means that negative result is approximately 7-fold more likely in a cat without than with FP. It is commonly accepted that LR- should be below 0.1 to be diagnostically useful i.e. negative result should be at least 10-fold less likely to occur in a diseased than heathy individual [33]. On the other hand, plenty of tests of worse accuracy are commonly used in veterinary medicine, to mention only DGGR-lipase assay which at a cut-off value of 26 U/L shows LR- equal to 0.4 (calculated from the formula: LR- = (1-Se) / Sp = (1–0.667) / 0.786; based on the figures reported in the study of Oppliger at al [13].). Detailed relationship between a pre-test probability of disease, LR, and a post-test probability of disease may be calculated by transformation of probabilities into odds [33, 34] or from very convenient Fagan’s nomogram [35, 36]. Nevertheless, the rule of thumb says that the negative test result whose LR- is 0.1 reduces pre-test probability by roughly 45% while LR- of 0.2 reduces it by roughly 30%. CBC model is somewhere in between. However, in fact it is safer to stick to the upper 95% confidence limit of LR- as tests usually perform worse in practice than they do in the population on which their accuracy has been assessed. Hence, when we assume that LR- is indeed closer to 0.4 and the pre-test probability of FP in a cat presented to the veterinary clinic is 30% if this cat tests negative in CBC model its post-test probability will be roughly 15%. Whether it is enough to justify practical use of CBC model is debatable. However, it is indisputable that applying CBC model costs nothing when CBC has already been done. On the other hand, we should be very cautious when using the CBC model as a confirmatory test. Even though, LR+ is very high (infinite) at certain cut-off values (Table 4) 49 cats used to determine Se (i.e. those classified as at increased risk of FP) were only moderately likely to have FP (as discussed in the next paragraph). Therefore, e.g. the cat with WBC ≥18 G/L, BNC ≥ 0.27 G/L, and EC < 0.3 G/L (the highest possible result of the CBC model) does not necessarily has FP. This cat simply appears to have an acute inflammatory reaction associated with at least one of the two tests (fPLI or AUS) positive for FP. Obviously, this result should make a practitioner examined this cat for FP using more specific methods, there are however many other diagnoses still probable in this cat.

The aforementioned limitation in the interpretation of positive results of the CBC model is associated with the fact that accuracy measures are of any value only if they have been estimated on the population of animals whose true heath status has been correctly determined [25, 37]. In our study we used two independent tests to diagnose FP – fPLI and AUS, at a cut-off value of 3.5 μg/L and at least one ultrasonographic feature of FP, respectively. We had to make a decision of how to handle inconsistent results of the two tests as the agreement between these test has been shown to be low [38, 39]. The problem of inconclusive classification of animals in the studies investigating diagnostic accuracy of a test when no 100% accurate gold standard exists has been thoroughly reviewed [40]. Even though many approaches exist, all of them aim to eventually assign inconclusive individuals to one of the two groups (healthy or diseased) as evaluation of diagnostic accuracy is only possible when animals are classified according to a dichotomous manner. On the other hand, elimination of cats with inconsistent results would create two artificial groups of cats, extreme in terms of the likelihood of FP, with a huge gap between them. This would make these groups differ not only in terms of having FP but also in a general condition and many other features remaining beyond our control. As a result the accuracy measures of the CBC model would be falsely inflated. In our study we decided to apply fPLI and AUS in a parallel fashion which meant that any positive result indicated FP [25]. Diagnostic accuracy of each of these two tests at cut-off values applied in our study has so far been estimated several times before. In terms of fPLI Se and Sp were estimated at 61% (CI 95%: 36%, 82%) and 55% (CI 95%: 39%, 70%), respectively, on 60 cats [13], 65% (CI 95%: 43%, 83%) and 63% (CI 95%: 26%, 90%), respectively, on 31 cats [12]. In an another study [14] Se was 74% for mild and 82% for marked FP, while Sp was 74%. In terms of AUS Se was 84% (CI 95%: 60%, 97%) and Sp was 75% (CI 95%: 48%, 93%) on 35 cats [16]. Assuming Se and Sp of 65% and 65%, respectively, for fPLI and 85% and 75%, respectively, for AUS, parallel testing procedure yields Se of 95% and Sp of 49%. Then, assuming pre-test probability of FP of 50%, the cat negative in both tests and on this basis considered as being at very low risk of FP in our study was 95% likely to be truly free from FP (therefore we referred to this group as cats at very low, not simply low, risk of FP). The cat positive in at least one of these tests was 65% likely to truly have FP, while a cat positive in both tests was 85% likely to truly have FP. Our CBC model is a screening test so we want to trust its negative result more than its positive result as the latter one will be further verified by tests more specific for FP. Therefore, in the study population we are more concerned with correct classification of cats as free from FP than as affected by FP. On the other hand, low Sp of a reference procedure tends to falsely reduce Se of an index test [41], so Se of the CBC model may be even higher than our estimations.

The most important limitation of our study is associated with the precision of CBC measurements used for the model development. While WBC was measured by modern automatic hematologic analyzers of high quality whose results are very likely to be highly repeatable, differential counts were determined manually by counting only 100 subsequent leukocytes. Such a method is prone to both human errors and random variability. For instance, CI 95% for BN% of 1% estimated from 100 cells is from 0.2% to 5.4% [42], which markedly exceeds the cut-off value indicated by our analyses. Therefore, routine counting of 200 or even 300 leukocytes appears to be a reasonable recommendation (CI 95% narrows down then to 0.1% to 2.8% and 0.1% to 1.9%, respectively), although it would considerably extend the turnover time of blood samples. On the other hand, manual reviewing of blood films is the only method allowing to include band neutrophil count in the result. Our study substantiates the practical usefulness of estimating band neutrophil percentage and counts, which is especially important given the growing popularity of in-clinic instrumental hematologic analyzers. Another important downside of our analysis is the fact that we did not include the information on toxic changes in neutrophils. It was so since the evaluation of toxic neutrophils was not routinely provided by the veterinary laboratory in which our samples were examined. Toxic neutrophils are an important feature of CBC in acute inflammation caused by an enhanced turnover rate of neutrophils and incomplete neutrophil maturation in the bone marrow [26, 31]. Including this variable might have improved performance of the CBC model and it certainly warrants further investigation.

## Conclusions

The combination of CBC measurements is an immediately available and fairly accurate screening method allowing to decide which cats presented with lethargy and anorexia have increased risk of FP.

## Methods

This retrospective cross-sectional study comprised 73 cats presented to three veterinary clinics located in the Central Poland in years 2014–2020 and screened for FP using the commercial feline pancreatic lipase immunoreactivity (fPLI) assay and abdominal ultrasonography (AUS). Cats were enrolled in the study if their owners declared that a cat: 1) had lethargy and reduced appetite or anorexia for at least two previous days; 2) did not have any known chronic diseases; 3) had not been diagnosed with FP before; 4) had not been treated with glucocorticosteroids for a preceding month. All cats were examined by a specialist in small animal medicine. The blood sample was collected for a routine blood analysis and fPLI quantification. fPLI was measured [μg/L] using ELISA based on monoclonal antibodies (Spec fPL™) in IDEXX Laboratories GmbH (Ludwigsburg, Germany). According to the manufacturer’s recommendations fPLI was considered normal when ≤3.5 μg/L and elevated when > 3.5 μg/L. AUS was performed by a veterinary radiologist with state-of-the art devices (MyLab 25 Gold, Esaote, Italy and HM70A, Samsung Electronics Ltd., UK). FP was ultrasonographically diagnosed if at least one of the following features was detected: pancreatomegaly (width of the pancreas > 10 mm), irregular margins of the pancreas, hypoechoic parenchyma of the pancreas, hyperechoic surrounding mesenteric or fat, and peripancreatic free fluid. Hyperechoic pancreatic parenchyma indicative of fibrosis was also considered diagnostic for FP [5, 10, 16]. Other diagnostic tests (thoracic radiography, echocardiography) were performed if necessary to make a definitive diagnosis.

Results of the two diagnostic tests used for detection of FP were interpreted according to a parallel manner [25] i.e. the risk of FP was considered as very low only when fPLI was normal (≤ 3.5 μg/L) and the appearance of the pancreas in AUS was normal. Otherwise, a cat was found to be at increased risk of FP.

The blood analysis was performed in a commercial veterinary laboratory and included routine complete blood count (CBC), alanine aminotransferase (ALT) and alkaline phosphatase (ALP) activity, total protein (TP) and total bilirubin (TB) concentration as well as urea and creatinine concentration. Routine blood analysis was performed using Abacus Vet5 Hematology analyzer (Diatron MI Zrt., Hungary), Mythic 18 Vet (PZ Cormay S.A., Poland) and automatic photometric clinical chemistry analyzer BS-800 (Mindray Medical Poland). The differential leucocyte count was performed on thin and smooth peripheral blood smears stained with the May-Grünwald-Giemsa reagent (Adamed Pharma S.A., Poland). The smears were examined with a light microscope (Primo Star, Zeiss, Germany) under 100x magnification by a qualified technician and 100 nucleated cells were classified into six subpopulations: band neutrophils, segmented neutrophils, eosinophils, basophils, monocytes, and lymphocytes. Basophils were not included in further analyses as they were very rarely observed in manual blood smears and their correct identification was questionable.

Four clinical conditions were identified in cats using hematologic and biochemical criteria: anemia when Ht was < 27%; hemoconcentration when i) Ht was > 45% and TP was > 80 g/L or ii) urea concentration was > 12 mmol/L at creatinine concentration <  250 μmol/L; suspected acute kidney injury when creatinine concentration was > 250 μmol/L and urea concentration was > 12 mmol/L, and suspected hepatopathy when ALT was > 200 U/L (without concurrent hyperthyroidism) or TB was > 17 μmol/L (without concurrent anemia) [43]. Diabetes mellitus was diagnosed based on polyuria and polydipsia in the medical history and fructosamine concentration > 400 μmol/L, hyperthyroidism based on total thyroxine concentration > 65 nmol/L, and neoplasms based on chest radiography, AUS, and ultrasound-guided fine needle biopsy. Fever was defined as rectal temperature > 39.5 °C. The presence of abdominal pain was subjectively assessed by a clinician.

Seven CBC measurements were analyzed in this study: WBC, total neutrophil count, band neutrophil count (BNC), eosinophil count (EC), monocyte count (MC), lymphocyte count (LC), and neutrophil-to-lymphocyte ratio (NLR).

No ethics commission approval for this study was required according to Polish legal regulations (the Act on the Protection of Animals Used for Scientific or Educational Purposes of 15 January 2015) as only routine diagnostic procedures essential given the clinical status of the cat were performed and the study was purely analytical. Nevertheless, an informed consent of each cat’s owner for participation in the study was obtained.

Numerical variables were presented as the median, interquartile range (IQR) and range, and compared between two groups using the Mann-Whitney U test. Categorical variables were presented as a count (n) and percentage of the total number of animals in a given group. First, CBC measurements were transformed into dichotomous variables. The optimal cut-off value for dichotomization was determined using the highest Youden’s index (J) criterion [24]. Then, the relationship between the categorized CBC measurements and the risk of FP was investigated using the maximum likelihood G-test or Fisher’s exact test if the expected count in any cell of the two-by-two table was < 5, and was expressed as the crude odds ratio (OR_crude_).

CBC measurements which proved significantly linked (α = 0.05) to the risk of FP in the univariable analysis were offered to the multiple logistic regression [44] according to the following formula:

$$\mathrm{f}\left(\mathrm{P}=1\right)=\frac{1}{1+{\mathrm{e}}^{-\left({\mathrm{B}}_0+{\mathrm{B}}_{\mathrm{n}}\times {\mathrm{X}}_{\mathrm{n}}\right)}}$$

where e signified the Euler’s number (≈2.718), B_0_ was the constant, B_n_ were the regression coefficients of CBC measurements (X_n_) entered in the initial model and evaluated using the backward stepwise elimination procedure. The magnitude of association between a CBC measurement and the risk of FP was expressed as the adjusted odds ratio (OR_adj_). The goodness-of-fit of the model was evaluated using the Hosmer-Lemeshow χ^2^ test (H&L χ^2^) and Negelkerke’s pseudo-R^2^ coefficient. Discriminatory potential of CBC measurements and logistic model based on CBC measurements (CBC model) in classifying cats into those with very low or increased risk of FP was assessed by computing the area under ROC curve (AUROC). AUROC was interpreted as follows: ≥90% – an excellent test, 80–89% – a good test, 70–79% – a fair test, < 70% – a poor test [24] and compared using the nonparametric method [45]. For the optimal cut-off value diagnostic sensitivity (Se), diagnostic specificity (Sp) as well as positive and negative likelihood ratios (LR+ and LR-) were calculated to evaluate diagnostic usefulness of CBC model. The 95% confidence intervals (CI 95%) for proportions and LRs were calculated using the Wilson score method and log method, respectively [46]. A significance level (α) was set at 0.05. All statistical tests were two-sided. The statistical analysis was performed in TIBCO Statistica 13.3 (TIBCO Software Inc., Palo Alto, CA, USA) and IBM SPSS Statistics 26 (IBM Corporation, Armonk, NY, USA).

All methods were carried out in accordance with relevant guidelines and regulations.

## Supplementary Information

**Additional file 1.** Numerical values of 7 complete blood count (CBC) measurements in 73 lethargic and anorectic and the cut-off values used for their categorization based on the highest Youden’s index (J).

## Data Availability

The database is available from authors on request.

## Abbreviations

α: Significance level
AUROC: Area under ROC curve
AUS: Abdominal ultrasonography
BNC: Band neutrophil count
CBC: Complete blood count
CI 95%: 95% confidence interval
DGGR: 1,2-o-Dilauryl-Rac-Glycero-3-Glutaric Acid-(6′-Methylresorufin) Ester
FP: Feline pancreatitis
fPLI: Feline pancreatic lipase immunoreactivity
H&L χ^2^: Hosmer & Lemeshow chi-square test
IQR: Interquartile range
LR+: Positive likelihood ratio (likelihood ratio of a positive result)
LR-: Negative likelihood ratio (likelihood ratio of a negative result)
NLR: Neutrophil-to-lymphocyte ratio
NPV: Negative predictive value
PPV: Positive predictive value
ROC: Receiver operating characteristic
Se: Diagnostic sensitivity
Sp: Diagnostic specificity
WBC: Total leukocyte count (white blood cell count)
