# Supplementary material for: Application of a complete blood count to screening lethargic and anorectic cats for pancreatitis
Source: BMC Vet Res. 2021 Dec 11;17:383. doi: 10.1186/s12917-021-03098-z (PMC8665532; doi:10.1186/s12917-021-03098-z)
Supplement: Supplementary file 1 — Additional file 1. Numerical values of 7 complete blood count (CBC) measurements in 73 lethargic and anorectic and the cut-off values used for their categorization based on the highest Youden’s index (J). [file 12917_2021_3098_MOESM1_ESM.docx]

Additional file 1. Numerical values of 7 complete blood count (CBC) measurements in 73 lethargic and anorectic and the cut-off values used for their categorization based on the highest Youden’s index (J)

| CBC measurements ^a^ | Risk of feline pancreatitis | | p-value^b^ | Youden’s index (J) (CI 95%) | Optimal cut-off value |
| --- | --- | --- | --- | --- | --- |
|  | Very low (n=24) | Increased (n=49) |  |  |  |
| Total leukocyte count (WBC) [G/L] | 11.7, 7.50 – 15.4 (3.20 – 32.1) | 12.8, 7.80 – 19.9 (2.00 – 36.4) | 0.280 | 30.5 (22.6, 38.5) | 18 |
| Total neutrophil count (TNC) [G/L] | 7.34, 4.91 – 12.0 (1.82 – 27.0) | 10.4, 6.21 – 15.6 (0.80 – 29.5) | 0.089 | 26.4 (17.5, 27.9) | 15 |
| Band neutrophil count (BNC) [G/L] | 0, 0 – 0.20  (0 – 1.45) | 0.29, 0 – 1.35  (0 – 8.71) | 0.002 | 40.5 (30.1, 50.9) | 0.27 |
| Lymphocyte count [G/L] | 2.47, 1.55 – 3.40 (0.47 – 4.99) | 2.10, 1.40 – 3.12 (0.55 – 7.00) | 0.518 | 19.6 (7.5, 31.8) | 2.2 |
| Eosinophil count [G/L] | 0.39, 0.20 – 0.65  (0 – 3.21) | 0.28, 0.06 – 0.56  (0 – 8.13) | 0.139 | 25.9 (13.9, 37.8) | 0.3 |
| Monocyte count [G/L] | 0, 0 – 0  (0 – 0.15) | 0, 0 – 0.05  (0 – 1.46) | 0.055 | 18.3 (11.1, 25.5) | 0.15 |
| Neutrophil-to-lymphocyte ratio (NLR) [1/1] | 3.57, 1.86 – 4.62 (0.79 – 31.7) | 5.17, 2.67 – 7.33 (0.67 – 19.0) | 0.063 | 30.1 (18.8, 41.4) | 4.7 |

^a^ reported as the median, interquartile range (IQR) and range in parentheses

^b^ the Mann-Whitney U test
